# Ultrafast Laser-Enabled 3D Glass Microchannel Reactors

**DOI:** 10.3390/s25237159

**Published:** 2025-11-24

**Authors:** Xiaolong Li, Jinxin Huang, Jian Xu, Ya Cheng

**Affiliations:** 1 Engineering Research Center for Nanophotonics and Advanced Instrument, School of Physics and Electronic Science, East China Normal University, Shanghai 200241, China; xlli@phy.ecnu.edu.cn (X.L.); 18955822963@163.com (J.H.); 2 XXL—The Extreme Optoelectromechanics Laboratory, School of Physics and Electronic Science, East China Normal University, Shanghai 200241, China

**Keywords:** continuous-flow synthesis, glass microchannel reactors, ultrafast laser-assisted etching, 3D microstructures, online monitoring

## Abstract

Microchannel reactors are among the most important tools used for high-performance continuous-flow synthesis. However, most microchannel reactors manufactured with conventional micromachining techniques are limited to two-dimensional (2D) planar geometries, which pose significant challenges for the custom production of three-dimensional (3D) architectures that offer superior microchemical performance. Using unique nonlinear optical effects of ultrafast lasers, hollow microchannel structures with 3D configurations can be flexibly created within transparent glass materials through either direct removal or subsequent chemical etching methods. This review provides an overview of typical fabrication techniques for 3D glass microchannel reactors based on ultrafast laser microfabrication, as well as their state-of-the-art advancements, including large-scale and high-precision manufacture of all-glass microchannels and the facile integration of online monitoring modules. Moreover, the applications of these fabricated microchannel reactors for various continuous-flow microchemical reactions are introduced. Ultrafast laser-enabled 3D glass microchannel reactors hold great potential for developing innovative and industrial-scale continuous-flow manufacturing processes in chemical engineering and pharmaceutical production.

## 1. Introduction

Continuous-flow chemistry is profoundly reshaping the landscape of fine chemical synthesis [1,2,3,4,5], pharmaceutical manufacturing [6,7,8], functional materials fabrication [9,10,11], medical diagnostics [12], environmental governance [13,14,15], and energy conversion [16,17,18], progressively emerging as a pivotal developmental direction in modern synthetic fields [19]. Compared to traditional batch reactors, continuous-flow systems offer an exceptional technological platform for hazardous reactions and rapid mixing processes due to their precise mass/heat transfer control, inherent process intensification safety features, and superior reaction selectivity [20,21,22,23,24,25]. Among these systems, microchannel reactors have become key enablers for high-efficiency continuous-flow synthesis. Their exceptionally high surface-area-to-volume ratio and precise fluid manipulation capabilities enable near-instantaneous heat transfer and highly efficient mass transfer, significantly enhancing reaction rates and selectivity [26,27,28,29,30]. However, current mainstream microreactor manufacturing technologies—whether lithography [31] and anisotropic etching/deep reactive ion etching (DRIE) [32] for silicon or glass substrates, or soft lithography [33,34], hot embossing [35,36,37], and injection molding [38] for polymers—are inherently planar processes. When handling rapid reaction systems or high-viscosity fluids, existing 2D microchannel reactors face certain limitations. Short diffusion distances conflict with the longer residence times required for reactions, while longer channels increase flow resistance and pressure drop [39,40], affecting system stability and efficiency. Although the mixing performance of microchannel reactors can be enhanced by applying external fields such as acoustofluidics [41], the limitations mentioned above still restrict the overall performance of most 2D microreactors. In contrast, 3D microchannel reactors enhance hydrodynamic performance and system integration by expanding spatial dimensions. By repeatedly folding laminar flows to accelerate diffusion processes [42,43] and inducing forced secondary flows (e.g., Dean vortices [44,45,46], chaotic advection [47,48]), 3D microchannels exhibit superior potential for rapid and efficient fluid mixing. Furthermore, the 3D architecture enables vertical stacking and interconnection of channels, eliminating the constraints of 2D planar layouts to achieve higher spatial density of functional units, shorter fluid transfer paths, and reduced dead volumes. Notably, these attributes establish 3D microchannel reactors as ideal platforms for high-throughput applications—their vertical stacking capacity supports large-scale parallelized unit integration (e.g., multilayered reaction arrays), increases flux density per unit chip area, and enables more compact continuous-flow processing systems, ultimately facilitating enhanced sample processing or reaction steps per unit time and providing technical pathways for high-throughput scenarios such as drug screening and synthesis [49]. As a result, the flexible design and creation of microchannels in 3D space offer a promising avenue for advancing next-generation high-performance, multifunctional integrated microreactors. However, current approaches primarily rely on multilayer bonding techniques to fabricate 3D microreactors [50,51]. The cost and complexity of the process, along with technical limitations of multilayer bonding techniques—such as risks of interface failure, thermal stress issues, misalignment between layers, and spatial structural limitations—restrict its use in the precise manufacturing of high-performance 3D microchannel reactors.

In recent years, the rapid development of ultrafast laser micro/nano-processing technology has opened new pathways for the fabrication of 3D glass microchannels [52,53,54,55,56,57]. This technique relies on nonlinear optical interactions (such as multiphoton absorption and avalanche ionization) between ultrafast laser pulses and transparent dielectric materials, enabling spatially selective material modification within the glass with high resolution down to the sub-micron scale [58,59,60,61]. By accurately controlling the laser focus’s 3D motion and using optimized chemical etching protocols, it can effectively create embedded 3D microfluidic networks with almost any topological design [62,63]. The development of ultrafast laser microfabrication technology has effectively overcome the limitations of conventional micro/nanofabrication techniques in constructing complex, 3D stereoscopic fluidic structures. It should be noted that creating 3D microchannels with transparent crystalline materials beyond optical glasses—such as sapphire, YAG crystal, and quartz [64,65,66,67]—is technically feasible. However, most current commercial transparent microchannel reactors primarily use glass materials because of their cost-effectiveness and scalability. Empowered by high-performance optical glass substrates such as fused silica and borosilicate glass, this technology provides a highly flexible solution for the customized fabrication of high-performance 3D glass microchannels. This unique “inside-out” true 3D manufacturing paradigm enables the creation of complex, free-form geometrical channels within a single transparent glass substrate, improving fluid mixing efficiency and facilitating high-level integration of multifunctional components. This review outlines the fundamental principles and technological advancements in ultrafast laser microfabrication of 3D glass microchannels. It also showcases representative microreactor applications of these fabricated microchannels in high-throughput, high-performance continuous-flow synthesis for advanced pharmaceuticals and chemical products. Typical examples include the efficient chemical conversion of carbon dioxide (CO_2_) [68], on-chip ultraviolet (UV) photochemical synthesis of vitamin D3 (VD3) [69], and real-time spectroscopic monitoring during the on-chip synthesis of functional nanostructures [70].

## 2. Fabrication of Glass Microchannel Reactors Using Ultrafast Laser

**Figure 1:**
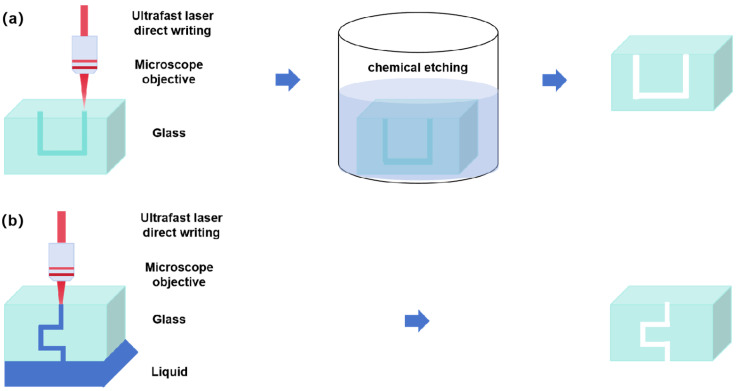
Representative methods for ultrafast laser fabrication of 3D glass microchannel structures. (**a**) Ultrafast laser-assisted etching; (**b**) Liquid-assisted ultrafast laser ablation.

Currently, two primary methods are used for the ultrafast laser fabrication of 3D glass microchannels [71,72,73]. One is ultrafast laser-assisted etching (i.e., ultrafast laser irradiation followed by selective chemical etching or ultrafast laser direct writing combined with subsequent chemical etching). As illustrated in Figure 1a, ultrafast laser direct writing is first used to create spatially selective modifications within the glass through nonlinear optical interactions [74,75,76,77,78]. Next, the laser-modified glass sample is placed in a container filled with chemical etchant. Due to the increased chemical reactivity of the laser-modified regions, these areas are selectively dissolved by the etchant, resulting in the final formation of a 3D glass hollow microchannel structure. In 2001, Marcinkevičius et al. pioneered the fabrication of microfluidic channels in silica glass using a femtosecond (fs) laser, establishing a novel pathway for the manufacturing of 3D glass microfluidic devices [74]. In 2004, Bellouard et al. employed fs laser irradiation followed by chemical etching to produce high-aspect-ratio open microchannels in fused silica [75]. The other is liquid-assisted ultrafast laser ablation (i.e., liquid-assisted ultrafast laser drilling). As depicted in Figure 1b, ultrafast laser direct writing is employed to induce ablation starting at the interface between the glass surface and a liquid medium. Introducing the liquid during laser ablation facilitates effective debris removal and stress management. Continuous laser writing enables the creation of 3D microchannels in glass without the need for additional chemical etching [79,80]. This technique enables efficient debris removal for high-aspect-ratio microchannels through the combined effects of plasma shockwaves and hydrodynamic forces at the glass–liquid interface [81,82,83,84,85]. In 2001, Li et al. achieved direct 3D hole drilling using fs laser pulses with the assistance of distilled water, demonstrating the feasibility of fabricating complex channels within transparent materials [81]. This technique delivers three key advantages through hydrodynamic effects: (i) flowing water removes ablated debris to minimize redeposition; (ii) water’s high thermal conductivity rapidly dissipates laser-induced plasma, reducing plasma absorption and scattering of subsequent laser energy; and (iii) suppression of thermal diffusion-induced microcrack formation enables direct internal microchannel preparation without post-processing. However, as the channel length grows, the viscous drag opposing debris ejection steadily increases, eventually stopping microchannel fabrication when debris cannot be evacuated. Furthermore, this technique shows relatively low efficiency for microchannel machining. Besides these two primary methods, the ultrafast laser fabrication of open microchannels on glass substrates, combined with ultrafast laser-induced glass microwelding, also offers a promising approach for all-glass microchannel fabrication. In this approach, an open microchannel is first fabricated through either ultrafast laser-assisted etching or ultrafast laser direct-write ablation. Then, the fabricated channel is welded to a planar glass to form a closed, all-glass microchannel via ultrafast laser-induced glass microwelding. Ultrafast laser-induced glass microwelding leverages plasma-induced melting at glass interfaces to achieve permanent bonding upon cooling [86,87,88,89,90]. By precisely regulating laser energy density (typically near the material modification threshold), this technique generates controlled micrometer-scale molten zones at bonding interfaces, achieving molecular-level diffusion bonding while preserving the structural integrity of the bulk material [91,92,93,94,95]. In 2005, Tamaki et al. reported the first glass-to-glass welding experiments via fs laser irradiation [86]. Sungil et al. combined fs laser-assisted etching with fs laser-induced welding to create highly reliable glass microfluidic devices, including microdroplet generators [87] and 3D magnetically active centrifugal micropumps [88]. Additionally, Wlodarczyk et al. developed a picosecond laser-induced welding technique for the rapid prototyping of various glass microfluidic devices [89,90]. Compared to traditional thermal compression bonding, the advantages of this approach include: (i) minimization of heat-affected zones to prevent deformation or collapse of microchannel structures due to thermal stress; (ii) compatibility with multilayer bonding after surface functionalization, enabling hermetic sealing without intermediate adhesive layers; and (iii) support for adaptive bonding of 3D freeform structures through region-selective welding in complex flow networks via dynamic focusing systems. However, this technology also has some limitations: a narrow process window with low tolerance for pulse energy deviations, and complex steps that require strict control over nonlinear dynamic characteristics in laser-material interactions. Additionally, production efficiency is limited by scanning strategies, making the fabrication of large-scale microreactors challenging. In this review, we focus on advancements in ultrafast laser-assisted etching for fabricating glass microchannels.

Ultrafast laser-assisted etching is a two-step technique for fabricating microchannels in glass [96]. First, ultrafast laser irradiation modifies the internal structure of the glass (Figure 1a), followed by selective etching with a specific chemical etchant to create hollow microchannels. Chemical etchant solutions, such as hydrofluoric acid (HF), potassium hydroxide (KOH), and sodium hydroxide (NaOH), are commonly used for selective chemical etching. However, the etching selectivity ratios (modified vs. unmodified regions) vary significantly among these etchants—approximately 100:1 for HF, 350:1 for KOH, and 600:1 for NaOH [75,97,98,99,100]. The physical mechanisms of ultrafast laser-assisted etching likely originate from laser-induced molecular restructuring. Ultrafast laser modification of fused silica decreases the average bond angle of the SiO_4_ tetrahedron (O_3_≡Si–O–Si≡O_3_) from 114°, leading to a reduction in the bond angle. This distortion in the oxygen atom’s valence electron structure enhances its chemical reactivity. Compared to unmodified regions, this densified structure in glass exhibits higher etching rates in acidic or hot alkaline environments, where the etchant dissolves material into soluble or volatile compounds to form microstructures [101]. Hnatovsky et al. demonstrated that the selective etching of fs-laser-modified fused silica strongly depends on the polarization direction of the laser beam [102,103]. Femtosecond laser irradiation under specific conditions creates nanograting-like structures in glass, which significantly influence the subsequent chemical etching process. When the laser writing direction is perpendicular to the polarization direction of the incident laser beam, the nanograting structures align along the writing direction, facilitating etchant penetration and accelerating the etching process in the laser-written regions [104,105,106,107,108]. Regarding the formation mechanism of nanograting structures, a variety of physical models (e.g., plasmon-photon interference, a transient nanoplasmonic model, and a self-trapped excitons model) have been proposed; detailed information can be found elsewhere [109,110]. Ultrafast laser-assisted etching has emerged as a cutting-edge method for fabricating high-performance glass microchannel reactors, owing to its unique advantages. Firstly, the minimal heat-affected zone of ultrafast lasers, combined with the smoothing effect of chemical etching, enables the fabrication of channels with submicron-level surface roughness. Secondly, its 3D processing capability allows for the free construction of arbitrarily complex geometric shapes (such as curves, intersections, and stacks) of microchannel networks within glass, breaking through the planar limitations of traditional photolithography and etching. Additionally, this technology exhibits broad applicability to various optical glasses (such as fused silica and borosilicate glass). Meanwhile, its maskless direct-writing characteristic simplifies the process flow, significantly enhances design flexibility, and achieves submicron-level high processing accuracy and resolution. In summary, ultrafast laser-assisted etching provides a powerful and flexible manufacturing platform for realizing glass microchannel reactors with high surface quality, complex three-dimensional structures, and high precision. It should be noted that ultrafast laser-assisted etching still faces significant challenges in fabricating large-scale, high-precision glass microstructures, particularly in the high-precision processing of centimeter-thick glass samples and the uniform etching of long channels. On the one hand, when processing centimeter-thick glass samples, low numerical aperture objectives with large working distances are often used for focusing, thereby reducing the axial resolution of laser processing. In particular, the cross-section of a focused ultrafast laser beam at the focus spot is typically ellipsoidal with a high aspect ratio, resulting in low axial resolution that impedes high-precision fabrication of complex structures in deep-depth applications. Meanwhile, the processing effects of laser focusing at different depths vary significantly due to spherical aberration [111,112], making it difficult to achieve depth-independent, high-precision three-dimensional processing. On the other hand, etchant diffusion proceeds inward from channel openings, resulting in longer etching times at the ends compared to the center, which produces significant tapering effects in long microchannels.

**Figure 2:**
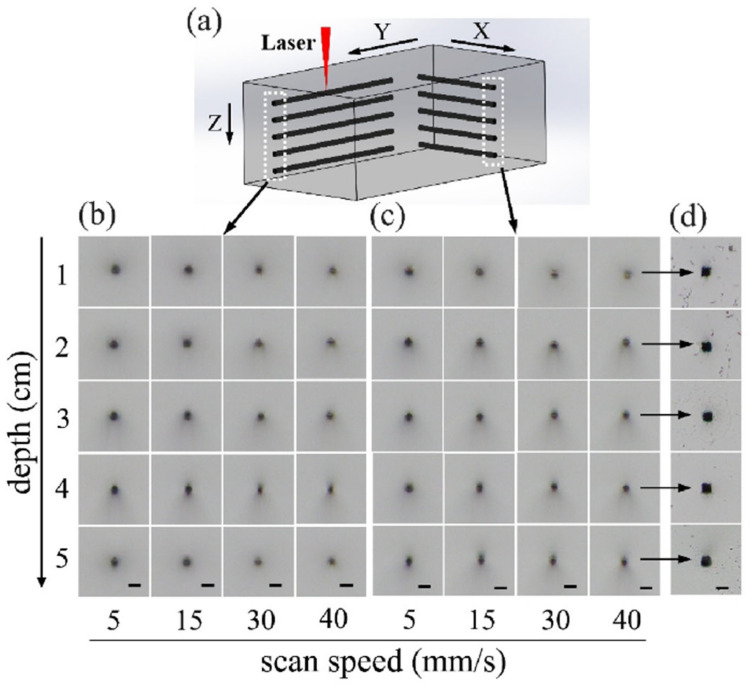
Depth-insensitive ultrafast laser direct writing in a thick fused silica glass sample. (**a**) Schematic of laser-inscribed lines within fused silica glass along the X and Y directions; Cross-sectional optical micrographs of the lines written along the (**b**) Y and (**c**) X directions; (**d**) Cross-sectional optical micrographs of hollow microchannel structures produced by chemically etching the inscribed lines in the last column of (**c**). Scale bar: 25 μm. Adapted from Ref. [113].

To address these challenges, it is necessary to develop new methods for high-performance laser-assisted etching. Recently, a technique for depth-insensitive and polarization-insensitive ultrafast laser direct writing has been proposed, which enables the generation of spatially symmetric spherical focal spots and exhibits a remarkable aberration-free processing capability [113]. With this technique, a series of lines was fabricated within fused silica at depths of 1, 2, 3, 4, and 5 cm below the glass surface, with scanning speeds set at 5, 15, 30, and 40 mm/s, respectively. In the experiment, an ultrafast laser source with a central wavelength of 1030 nm, tunable pulse widths ranging from 0.19 ps to 10 ps, and a maximum pulse energy of 1 mJ was used. For microstructure fabrication in glass, the laser repetition rate was set to 200 kHz, and the output power was controlled with a variable attenuator. The laser beam was first collimated and reduced to a 2 mm diameter using a telescope system, then directed through an acousto-optic modulator. Next, the beam was expanded with a beam expander and finally focused into the sample (a 55 mm-thick fused silica cube) using a 5× objective lens with an NA of 0.14. Figure 2a shows a 3D schematic of ultrafast laser writing in glass along the Y- and X-directions under identical conditions, while Figure 2b,c display the corresponding cross-sectional micrographs after laser processing with different parameters. As shown in Figure 2d, the cross-sectional shape and dimensions of the fabricated microchannels stay consistent despite changes in the writing depth. This confirms the depth-insensitive nature of the interaction between the ultrafast laser and fused silica. Furthermore, laser-induced selective etching using this technique is polarization-independent, overcoming the polarization sensitivity constraint inherent in conventional fs laser-assisted etching [114]. This technique enables the creation of 3D complex, high-precision glass structures at depths of up to several millimeters within glass substrates or in free-standing form.

**Figure 3:**
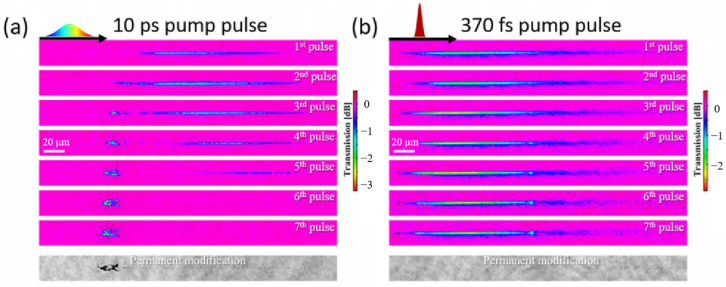
Transverse pump-probe microscopy in the multi-pulse interaction regime. The transient transmission images and corresponding static micrographs for material modifications were shown for the 10 ps (**a**) and 370-fs pump pulse (**b**). Scale bars: 20 μm. Adapted with permission from Ref. [120].

To understand the physical processes involved in depth-insensitive ultrafast laser direct writing, fs laser pump-probe shadowgraph imaging was employed to observe the generation and evolution of plasma at the focal position inside glass. The fs laser pump-probe shadowgraph imaging technique enables the visualization of plasma generation and evolution dynamics, which is beneficial for elucidating the physical mechanisms underlying the interaction between ultrafast lasers and glass materials [115,116,117,118]. In this technique, the fs laser beam is split into two paths using a beam splitter. The pump beam, after an extra-cavity pulse compressor has controlled its pulse width, is focused inside the fused silica to induce plasma. The probe beam propagates perpendicularly through the plasma region and is then recorded by a CCD camera. Owing to the strong absorption of the probe light by the plasma, areas corresponding to the plasma distribution appear as distinct shadows in the CCD-recorded images. An optical delay line in the probe beam path allows adjustment of the optical path difference between the probe and pump beams, enabling the capture of the plasma evolution at different time delays. Figure 3 presents transient absorption images captured at the time delay corresponding to the peak plasma density for both a 10 ps laser pulse and a 370-fs laser pulse, generated within fused silica under different numbers of pulses. The key observations were as follows: For the 10 ps laser pulse, as the number of pulses increased, the high-density plasma region shifted in the direction of laser incidence. Upon reaching seven pulses, a permanent modification region formed. Crucially, the spatial morphology of this modification exhibits close correspondence with the plasma distribution, as shown in Figure 3a. For the 370-fs laser pulse, under different pulse number conditions, the plasma consistently exhibited a longitudinally elongated morphology without a significant positional shift. Furthermore, no detectable material modification was observed after irradiation with seven pulses, as shown in Figure 3b. These phenomena could be attributed to the combined effects of reduced self-focusing, diminished pre-focal energy depletion, and shortened plasma defocusing events. The results revealed that an incubation effect may play a critical role during the interaction between picosecond laser pulses and the glass substrate. Due to the presence of defect centers, structural rearrangements, and mechanical damage following laser irradiation, the electron binding energy in irradiated glass regions was significantly reduced compared to the pristine state. This may facilitate photoionization activation in modified zones at lower intensity thresholds, promoting rapid generation of free electrons by the leading edge of subsequent pulses. The remaining pulse energy further enhanced collisional heating of free electrons via the inverse bremsstrahlung mechanism. When electron kinetic energy exceeds the bandgap energy of irradiated regions (~9 eV for pristine fused silica), avalanche-like multiplication of free electrons in the conduction band is triggered. Since the sustained electric field of picosecond pulses provides sufficient acceleration for directly photoionized free electrons, their interaction with irradiated zones is highly susceptible to pre-existing intrinsic defects inherited from prior pulses. Ultimately, through cumulative positive feedback arising from material transformations during multi-pulse interactions within the picosecond domain, highly selective spatial energy concentration is achieved [119,120].

**Figure 4:**
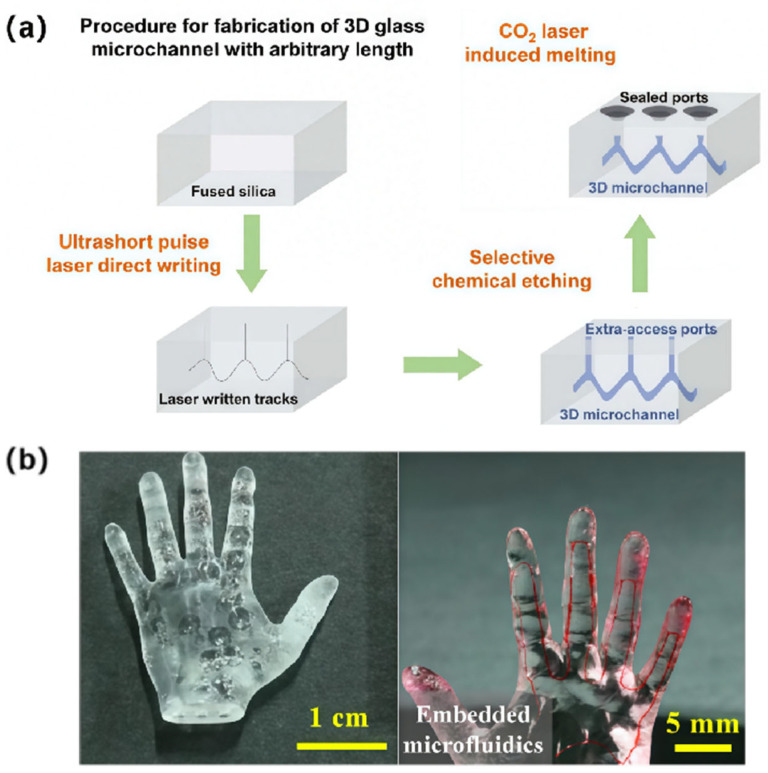
Ultrafast laser-assisted etching combined with CO_2_ laser-induced in situ sealing for fabricating arbitrarily long and uniform 3D microchannels inside glass. (**a**) Schematic of the 3D glass microchannel fabrication procedure; (**b**) 3D glass hand structure embedded with “vascular” microchannels: after sealing (left), microchannels filled with red ink (right). Adapted with permission from Ref. [121].

Meanwhile, to address the typical tapering issues in the fabrication of long and uniform microchannels inside glass substrates during the etching process, a technique based on ultrafast laser-assisted etching combined with CO_2_ laser-induced in situ glass sealing has also been developed [121]. As illustrated in Figure 4a, the fabrication process consists of two key steps: extra-access-hole-enhanced laser-assisted etching and CO_2_ laser-induced hole sealing. First, extra-access holes are properly introduced alongside the ultrafast laser-written microchannel patterns during the laser writing process. These holes serve to reduce etching time and enhance the precision and uniformity of the microchannels. Subsequently, a defocused CO_2_ laser beam irradiates the extra-access holes, inducing localized melting of the glass surface and forming a molten glass phase. Then, driven by surface tension and thermal gradients, this molten phase flows towards the center of the extra-access holes. As the laser irradiation time increases, the extra-access holes progressively diminish in size and are ultimately sealed by the solidified glass, forming an impermeable plug. By regulating the laser power, irradiation time, and defocusing distance, the thickness and depth of the sealing layer can be precisely controlled. Crucially, unlike conventional bonding and assembly techniques, this method imposes no constraints on the surface roughness of the glass, thereby facilitating the monolithic fabrication of large-scale, high-precision glass microchannels. In principle, as long as the working distance of the ultrafast laser processing stage permits, this technique can fabricate complex 3D microchannel networks with almost unlimited length. With this technique, a 3D glass hand structure with a whole size of several centimeters and embedded free-form microchannels was fabricated, as shown in Figure 4b. In the experiment, 3D microstructure fabrication was performed within the sample substrate using an ultrafast laser with a central wavelength of 1030 nm, a pulse width of 4 ps, and a repetition rate of 250 kHz. The laser beam was focused through an objective lens with an NA of 0.3 and scanned at a speed of 30 mm/s. To verify the sealing integrity of the internal vascular-like microchannel network, catheters were inserted through three incised openings on a wrist cross-section, and red ink was injected. The results showed that no leakage was observed in this structure, confirming that this technique is applicable to glass microchannels with complex surfaces and configurations.

## 3. Applications of Ultrafast Laser-Enabled Glass Microchannel Reactors

The latest advancement in ultrafast laser 3D microfabrication in glass, particularly the techniques of depth-insensitive and polarization-insensitive laser processing of thick glass substrates, as well as CO_2_-laser-assisted sealing of extra-access holes in glass, has provided new pathways for the customized fabrication of high-performance, large-sized 3D glass microchannels. This advancement has enabled the controllable manufacture of complex microchannels with high aspect ratios, thereby developing an ultrafast laser 3D printing technology for glass microchannel reactors that is suitable for industrial-scale production, offering high resolution and throughput. The manufactured glass microchannel reactors feature precisely designed 3D internal channels and large-capacity liquid storage areas, which have proven effective in solid–liquid reactions [122], gas–liquid reactions [68], deep-ultraviolet photochemical synthesis [69], and synthesis with online monitoring [70], offering technical support for the iteration and scale-up of various high-performance continuous-flow synthesis processes.

### 3.1. Continuous-Flow Solid–Liquid Reactions

**Figure 5:**
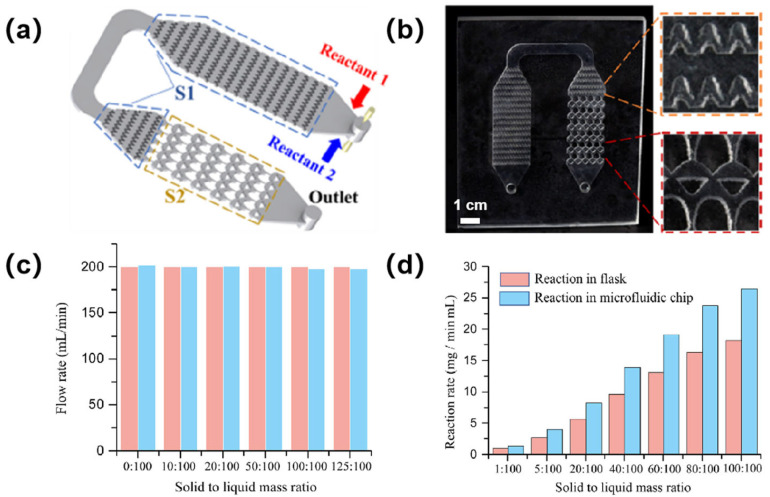
Ultrafast-laser-enabled glass microchannel reactor for solid–liquid reactions. (**a**) Schematic of the microchannel reactor; (**b**) Photos of the microchannel reactor (the microchannel reactor has dimensions of 90 mm × 75 mm × 6 mm, with the feature size of segment S1 measuring 16 mm in width and 1 mm in height); (**c**) Comparison of flow rate between set values and experimental data; (**d**) Comparison of reaction rates between microchannel reactor synthesis and flask synthesis systems. Adapted with permission from Ref. [122].

In continuous-flow solid–liquid reactions, a high volumetric fraction of solid reactants typically increases fluid viscosity, leading to hindered rapid transport and uniform mixing within microchannels, with potential channel clogging observed [123,124,125]. This issue could be improved by using an ultrafast laser-enabled 3D microchannel reactor [122]. As illustrated in Figure 5a, the microchannel reactor was composed of two functional segments: segment S1 was incorporated with a 3D vertical flow-splitting design (where the fluid was periodically compressed to amplify solid–liquid contact area), while segment S2 was integrated with a transversely arranged 5 × 6 array of 2D mixing units optimized through topology based on spline interpolation curves, enabling high mixing efficiency of the microchannel reactor. The microchannel reactor fabricated via ultrafast laser-assisted etching is displayed in Figure 5b, alongside microscopic views of its mixing units. To validate the performance of the microchannel reactor, an integrated flow synthesis system with UV-vis spectroscopic monitoring was constructed. Experimental results demonstrated that the actual flow rates of high-viscosity suspensions closely matched the set values (Figure 5c), and stable throughput was maintained throughout the 3D geometry without clogging. The successful synthesis of gadopentetate dimeglumine was achieved through solid–liquid phase reactions using this microchannel reactor. Reaction rate comparisons (Figure 5d) revealed a significant advantage of the microreactor: At a solid-to-liquid mass ratio of 5:100, a rate of 4.02 mg·mL^−1^·min^−1^ was achieved, representing a 46.7% enhancement over the conventional flask system (2.74 mg·mL^−1^·min^−1^). The result verified that the 3D glass microchannel provided a highly efficient platform for solid–liquid reactions, as shear-induced mixing effects were significantly enhanced.

### 3.2. Continuous-Flow Gas–Liquid Reactions

**Figure 6:**
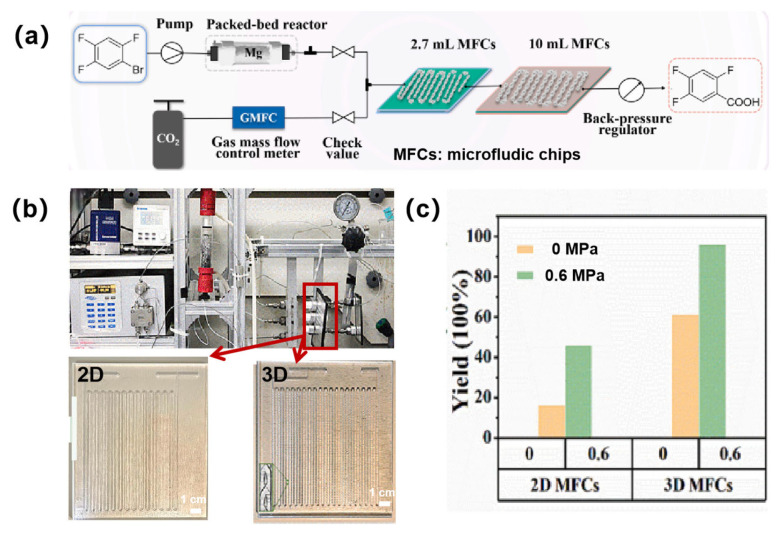
Ultrafast-laser-enabled glass microchannel reactor for gas–liquid reactions. (**a**) Schematic of a constructing continuous-flow gas–liquid reaction platform; (**b**) Photos of the gas–liquid reaction continuous-flow synthesis setup and the equipped 2D and 3D microchannel reactors (i.e., microfluidic chips, MFCs); (**c**) The yield comparison using 2D and 3D MFCs under 0 and 0.6 MPa. Adapted with permission from Ref. [68].

The conversion of CO_2_ into value-added chemicals was recognized as crucial for reducing fossil fuel dependence and achieving carbon neutrality goals [126,127]. However, challenges including the low reactivity of CO_2_, poor solubility in organic solvents, and inefficient gas–liquid mass transfer were identified as contributors to sluggish reaction kinetics [128,129,130]. The continuous-flow microreactor offers a novel approach for efficient CO_2_ conversion owing to its high specific surface area and exceptional thermal management capabilities [131,132,133]. A 3D glass microchannel reactor with Baker-transform structures was proposed and manufactured by ultrafast laser microfabrication. This reactor was further demonstrated to significantly expand the gas–liquid contact area, enabling efficient reactions at low Reynolds numbers [68]. As shown in Figure 6a, a continuous-flow gas–liquid reaction system was constructed using ultrafast-laser-enabled glass microchannel reactors. Experimental setups using the 2D and 3D glass microchannel reactors were established (Figure 6b), through which 2,4,5-trifluorobenzoic acid and diverse fluorinated carboxylic acids were successfully synthesized from Grignard reagents and CO_2_. Comparative analysis of gas–liquid carboxylation performance between 2D and 3D microchannel reactors under identical conditions (Figure 6c) revealed that the 2D microchannel reactor achieved product yields of 16% (0 MPa) and 46% (0.6 MPa) after 40 s carboxylation. In contrast, the 3D microchannel reactor with Barker-transform structure delivered substantially enhanced yields of 60% (0 MPa) and 96% (0.6 MPa) at identical residence time. A 44–50% yield improvement was demonstrated, attributed to an amplified gas–liquid interfacial contact area and intensified mass transfer. Notably, only a yield of 83% was achieved by conventional batch reactors after 4 h of processing at 0 °C, which underscored the transformative efficiency of the ultrafast laser-fabricated 3D microchannel platform for sustainable CO_2_ utilization.

### 3.3. On-Chip Continuous-Flow Synthesis with Real-Time Spectral Monitoring

**Figure 7:**
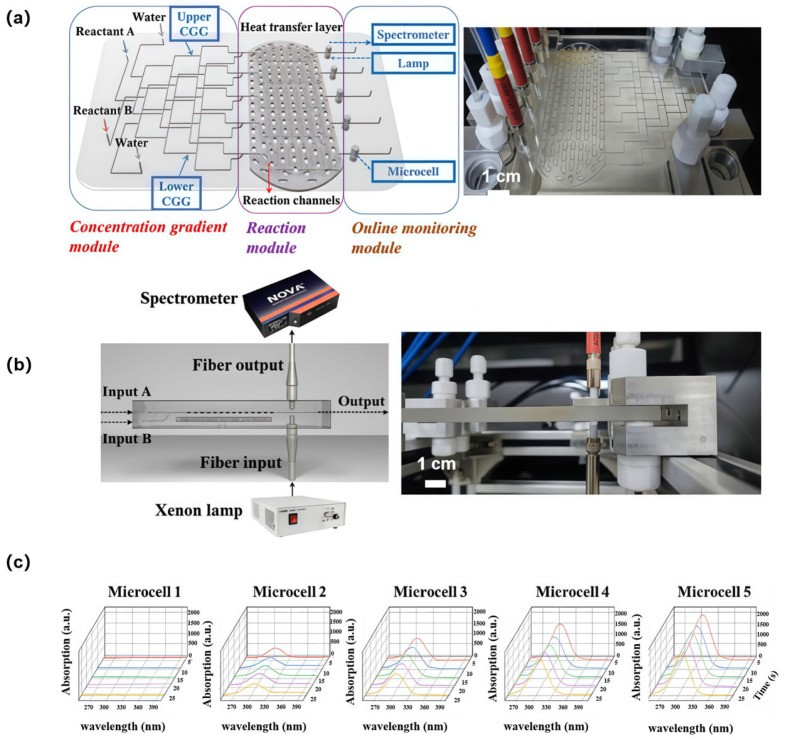
Ultrafast-laser-enabled glass microchannel reactor integrated with real-time spectroscopic monitoring functionality. (**a**) Schematic (left) and photo (right) of the microreactor (the size of microchannel reactor: 158 mm × 138 mm × 9 mm) consisting of a concentration gradient module, a reaction module, and an online monitoring module; (**b**) Schematic (left) and photograph (right) of the monitoring module integrated with a fiber optic probe; (**c**) Absorption spectra data collected from different microcells. Adapted with permission from Ref. [70].

To further enhance the functionality of the microchannel reactors, integrating probe-based sensing modules into microfluidic architectures for real-time monitoring of critical reaction parameters (e.g., reactant concentration, temperature) is necessary. This approach facilitates precise and autonomous continuous-flow synthesis while providing real-time feedback for process control [134,135,136,137]. To achieve this, a 3D glass microchannel reactor integrated with optical fiber probe arrays was manufactured using ultrafast laser microfabrication [70]. Real-time spectroscopic monitoring was achieved during on-chip continuous-flow synthesis. As shown in Figure 7a, the microreactor consisted of three modules: a concentration gradient module, a reaction module, and an online monitoring module. The concentration gradient module utilized 3D hydrodynamic manipulation to generate stepwise concentration profiles for rapid screening of optimized reactant concentrations. Five parallel 3D micromixing networks based on Baker’s transformation were integrated into the reaction module alongside heat-exchange channels, enabling efficient mixing under precise temperature control (−70 °C to 250 °C). Optical fiber probes were coupled with microcells in the inline monitoring module, enabling high spatiotemporal resolution and parallel monitoring of continuous-flow synthesis. Time-resolved spectra were collected from microcells using the online monitoring module (Figure 7b). Nearly invariant absorption peak positions and intensities were observed (Figure 7c), confirming the steady state of zinc nitrate (Zn(NO_3_)_2_) within the reactor. This result validated the high-resolution online monitoring capability of the system. Precise control over ZnO size and morphology was achieved by adjusting flow rates, reactant concentrations, and reaction temperatures in real time based on feedback from online spectral signals. This monolithically integrated microreactor features multifunctionality and high compactness. The real-time spectral database serves as a foundation for creating training material models, enabling the integration of artificial intelligence technology into continuous-flow synthesis for building closed-loop, controlled, smart “factory-on-a-chip” systems.

### 3.4. Automated Remote Synthesis

**Figure 8:**
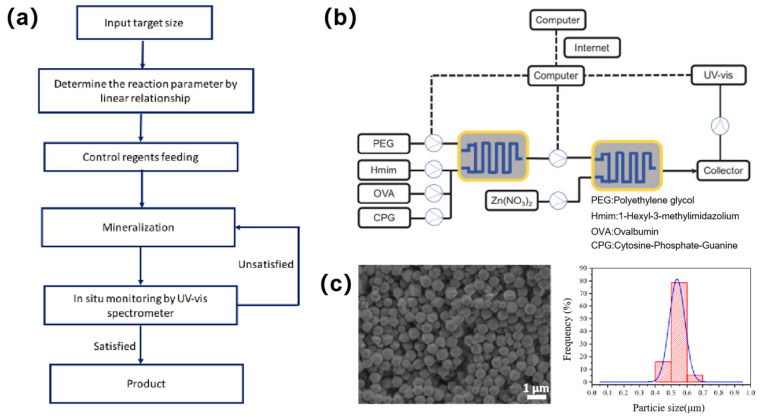
Remote automated synthesis in an ultrafast-laser-fabricated glass microchannel reactor. (**a**) A processing algorithm for automatic synthesis of PEG mineralized ZIF with controlled size; (**b**) Schematic of the remote automatic synthesis process for vaccine particles in the microchannel reactor; (**c**) SEM image of ZIF-8 particles automatically synthesized by the microchannel reactor (left) and static size distribution of particles (right). Adapted with permission from Ref. [149].

Advances in automated synthesis technology enabled the pharmaceutical industry to achieve significant progress in reducing research and development costs and accelerating drug development [138,139,140,141,142,143]. Polyethylene glycol (PEG)-mineralized zeolitic imidazolate frameworks (ZIF-8) bio-composites have demonstrated notable potential in drug delivery and vaccine carriers, while their automated and remote synthesis has been recognized as a critical breakthrough toward personalized therapies [144,145,146,147,148]. A fully automated synthesis platform based on an ultrafast laser-enabled 3D glass microchannel reactor was established [149]. Reagents were sequentially delivered into the system via peristaltic pumps, where reactants underwent mixing and reaction within the microchannel reactor, and then monitored in situ by UV-vis spectroscopy. A processing algorithm for automatic control (Figure 8a) was designed based on the linear relationship between PEG concentration and ZIF-8 particle size. Upon inputting the target particle size, the system automatically calculated the required PEG concentration, dynamically adjusted pumping parameters, and validated product formation via UV-vis absorption spectroscopy. Figure 8b illustrates the flowchart for remote automated synthesis. Successful internet-based control (transmission distance >20 km) for synthesizing uniform ZIF-8 particles under unattended conditions (Figure 8c) was demonstrated. Furthermore, by co-encapsulating ovalbumin (OVA) and adjuvant cytosine-phosphate-guanine (CpG), vaccine particles OVA@ZIF-8 and OVA-CpG@ZIF-8 were prepared. Flow cytometry confirmed their efficacy in significantly promoting dendritic cell activation and maturation. The developed remote automated synthesis platform offers a scalable technological route for the distributed manufacturing of personalized pharmaceuticals, such as cancer vaccines.

### 3.5. Ultraviolet Photochemical Continuous-Flow Synthesis

**Figure 9:**
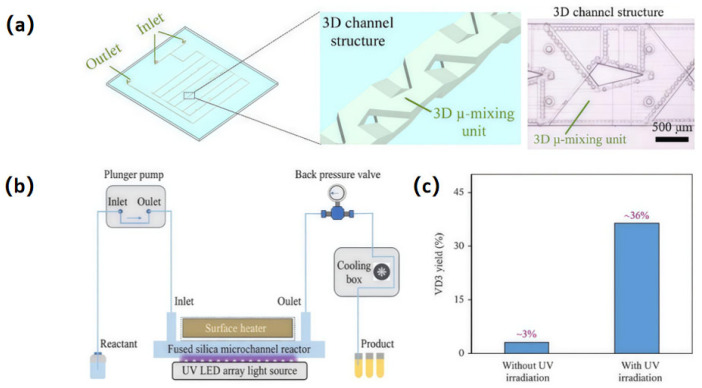
Continuous-flow UV photochemical synthesis in an ultrafast laser-fabricated glass microchannel reactor (the size of the microchannel reactor: 150 mm × 125 mm × 2 mm). (**a**) Schematic of the microchannel reactor integrated with 3D micromixing units (left), close-up view of the micromixing units (middle), and optical micrograph of the mixing unit (right); (**b**) Schematic of a continuous-flow UV photochemical synthesis system; (**c**) VD_3_ yield with and without UV irradiation (UV wavelength: 275 nm). Adapted from Ref. [69].

Although microchannel reactors hold significant potential for continuous-flow photochemical reactions, most commercially available glass microchannel reactors were manufactured from borosilicate glass, which suffers from poor transmittance in the short-wavelength ultraviolet (UV) spectrum, thereby hindering efficient photon utilization in deep UV photochemical synthesis [150,151,152,153,154,155]. A large-scale, high-transparency fused silica microchannel reactor has been developed, enabling efficient one-step UV photochemical synthesis of VD_3_ [69]. The combination of ultrafast laser-assisted etching and CO_2_ laser-induced in situ melting was used to fabricate a large-scale fused silica microchannel reactor integrated with 3D mixing units, as shown in Figure 9a. The unique designs of fabricated 3D mixing units enhanced fluid mixing in microchannels, including directions perpendicular to the horizontal plane, leading to improved UV light absorption by reactants and more uniform reactions within confined spaces. A continuous-flow UV photochemical synthesis system was further constructed based on the glass microchannel reactor, as depicted in Figure 9b. The system adopted a sandwich configuration sequentially comprising an LED light source, the fused silica microchannel reactor, and a surface heater. Through parametric optimization of UV-LED wavelength, fluid flow rate, temperature, and pressure, one-step continuous-flow production of VD_3_ was achieved with a yield of approximately 36% (Figure 9c). Compared to traditional continuous-flow UV photochemical synthesis, which often relies on high-pressure mercury lamps as light sources, UV-LED array-assisted photochemical synthesis offers a more efficient and stable approach for utilizing UV photon energy and maintaining long-term temperature control. This demonstrates the immense scale-up potential of the ultrafast laser-enabled glass microchannel reactors for industrial-scale UV photochemical synthesis applications. Furthermore, the fabricated fused silica microreactor has the potential to enable various catalyst-free UV photochemical synthesis methods due to its unique ability to operate under high-energy UV photon irradiation.

### 3.6. Synthesis of Ultrasmall Semiconductor Polymer Nanomaterials

**Figure 10:**
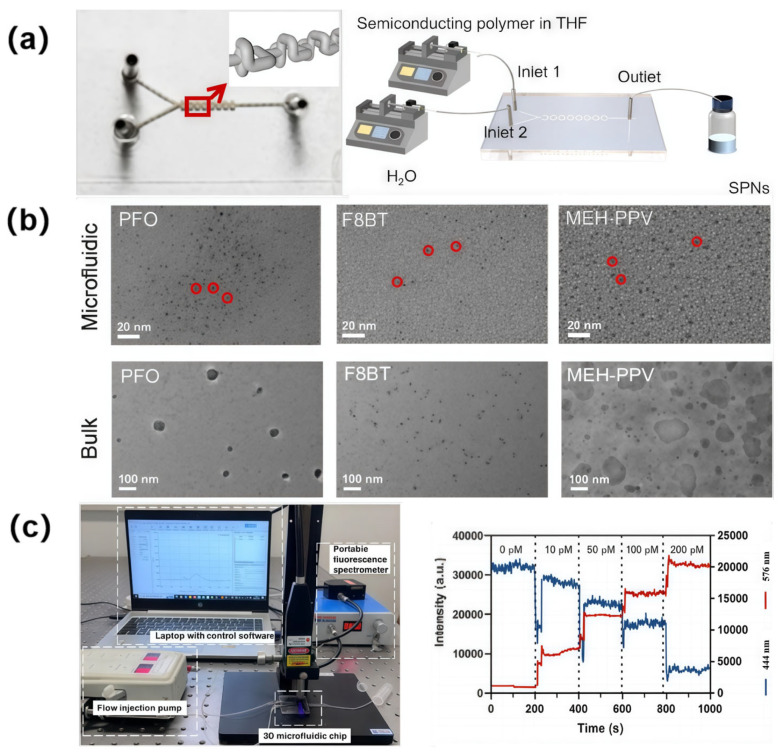
Controllable synthesis of ultrafine polymer nanoparticles in an ultrafast laser-fabricated 3D glass microfluidic chip (the size of the microfluidic chip: 30 mm × 40 mm × 3 mm). (**a**) Schematic of the 3D glass microfluidic chip (left) and the SPN synthesis system (right); (**b**) TEM images of SPNs (PFO/F8BT/MEH-PPV) synthesized in the microfluidic chip and the bulk (flask-based synthesis) system, respectively; (**c**) Photo of the fluorescence online monitoring system based on 3D microfluidic chip (left) and timing diagram for online detection of different concentrations of glucose (right). Adapted with permission from Ref. [163].

Semiconducting polymer nanoparticles (SPNs) offer several advantages, such as tunable optical properties, large extinction coefficients, high single-particle brightness, and excellent photostability, making them suitable for biomedical analysis, labeling, and imaging [156,157,158,159]. However, synthesizing SPNs through traditional methods often faces drawbacks such as low nanoparticle concentrations, poor size distribution uniformity, and difficulties in process control [160,161,162]. By using an ultrafast laser-fabricated glass microfluidic chip integrated with 3D micromixing units, ultrasmall SPNs have been synthesized at room temperature with precise regulation of reaction conditions [163]. The configuration of the glass microfluidic chip (left) and the SPN synthesis system (right) are shown in Figure 10a. After injection, reactants were subjected to repeated splitting and recombination within 3D micromixing units, enabling rapid and efficient mixing via alternately separated and concentrated flow paths. Three types of semiconducting polymer nanoparticles (PFO, F8BT, and MEH-PPV) were synthesized in the microchannel chip. These synthesized products were systematically compared with equivalents prepared via traditional nanoprecipitation in flasks with sonication. As demonstrated in Figure 10b, unlike flask-based synthesis, the glass microfluidic chip enabled microscopic control over reaction conditions. Uniform reagent mixing was achieved within milliseconds, resulting in reduced residence time distribution caused by inhomogeneous mixing. This approach was confirmed to significantly improve the size distribution and reproducibility of SPN through statistically validated particle characterization. The fluorescence-based online glucose monitoring system was illustrated in Figure 10c (left: reactor configuration; right: timing diagram). As glucose concentration increased, fluorescence intensity at 444 nm decreased progressively, while intensity at 576 nm increased, indicating a positive correlation. Automated mixing of dialyzed blood samples with fluorescent probes was implemented within the microfluidic chip, enabling immediate, dynamic, and continuous monitoring of blood glucose levels. This system was validated to assist diabetic patients in determining insulin doses through real-time tracking, establishing a foundation for personalized diabetes treatment strategies. This synthesis strategy, based on a 3D microfluidic chip, is promising for the scalable production of semiconducting polymer nanomaterials, demonstrating applicability in biosensing (e.g., tumor metabolite detection), controlled drug release systems, and photothermal therapy.

## 4. Summary and Outlook

The advancements in ultrafast laser microfabrication, particularly the development of depth-insensitive ultrafast laser processing and CO_2_-laser-induced in situ melting techniques, have enabled the customized fabrication of large-scale, high-throughput 3D glass microchannel reactors. By freely constructing complex 3D topological structures, the technology achieves seamless integration within a single glass substrate of curved flow channels, efficient mixing units, and other functional modules. Coupled with extreme environmental adaptability (withstanding temperature variations from −70 °C to 250 °C) and on-chip monitoring capabilities (fiber optic arrays), it provides an efficient platform for frontier applications, including solid–liquid reactions (46.7% increase in gadopentetate dimeglumine synthesis rate), gas–liquid mass transfer (96% yield in CO_2_ carboxylation), deep-ultraviolet photochemical synthesis (36% VD3 yield), and controllable nanoparticle synthesis (SPNs diameter < 5 nm). The distributed manufacturing facilitated by remote automated synthesis systems (with transmission distances exceeding 20 km) underscores the technology’s pivotal role in advancing continuous-flow chemistry toward high performance and intelligence. However, current challenges remain in achieving large-scale processing efficiency, controlling the roughness of internal walls of microchannels, and coordinating the design of multifunctional modules. Future development requires focused efforts on optimizing processing techniques to enhance efficiency and reduce roughness, establishing microchannel topology-mass transfer correlation models to guide specialized chip design, developing monolithic integration technologies for on-chip sensing and execution units, and implementing AI-driven machine learning for the autonomous optimization of channel parameters. Meanwhile, by further integrating technologies such as online spectroscopic in situ monitoring, AI algorithm design, and multiphysics digital twin technology, it is expected to achieve adaptive dynamic optimization of high-efficiency continuous-flow synthesis and intelligent decision-making for process parameters in microchannel reactors. This will ultimately accelerate the industrial application of intelligent continuous-flow manufacturing. As these directions progress, ultrafast laser-fabricated 3D glass microchannel reactors are poised to have a profound impact on the distributed manufacturing of personalized pharmaceuticals, carbon neutrality-oriented CO_2_ valorization, and AI-powered “factory-on-chip” systems. This promises transformative paradigm shifts in the chemical, biomedical, and energy materials industries.
